# Neuromechanical response to spinal manipulation therapy: effects of a constant rate of force application

**DOI:** 10.1186/s12906-016-1153-6

**Published:** 2016-06-02

**Authors:** François Nougarou, Isabelle Pagé, Michel Loranger, Claude Dugas, Martin Descarreaux

**Affiliations:** Université du Québec à Trois-Rivières, 3351, Boul. des Forges, Trois-Rivières, G9A 5H7 Québec Canada

**Keywords:** Spinal manipulation, Electromyography, Biomechanical phenomena, Dose-response relationship, Musculoskeletal manipulations

## Abstract

**Background:**

Neuromechanical responses to spinal manipulation therapy (SMT) have been shown to be modulated through the variation of SMT biomechanical parameters: peak force, time to peak force, and preload force. Although rate of force application was modulated by the variation of these parameters, the assumption that neuromuscular responses are modulated by the rate of force application remains to be confirmed. Therefore, the purpose of the present study was to evaluate the effect of a constant rate of force application in neuromechanical responses to SMT in healthy adults.

**Methods:**

Four SMT force-time profiles presenting different time to peak force and peak force, but with a constant rate of force application were applied on 25 healthy participants’ T7 transverse processes. Muscular responses were recorded through surface electromyography electrodes (T6 and T8 levels), while vertebral displacements were assessed through pasted kinematic markers on T6 to T8 spinous processes. Effects of SMT force-time profiles on neuromechanical responses were assessed using repeated-measures ANOVAs.

**Results:**

There was no main effect of SMT force-time profile modulation on muscular responses (*p*s > .05) except for the left T8 (*F* (3, 72) = 3.23, *p =* .03) and left T6 (*F* (3, 72) = 2.94, *p =* .04). Muscular responses were significantly lower for the lowest peak force condition than the highest (for T8) or second highest (for T6). Analysis showed that increasing the SMT peak force (and concomitantly time to peak force) led to a significant vertebral displacement increase for the contacted vertebra (*F*_T7_ (1, 17) = 354.80, *p <* .001) and both adjacent vertebras (*F*_T6_*(*1, 12) = 104.71, *p <* .001 and *F*_T8_ (1, 19) = 468.68, *p <* .001).

**Conclusion:**

This study showed that peak force modulation using constant rate of force application leads to similar neuromuscular responses. Coupled with previous investigations of SMT peak force and duration effects, the results suggest that neuromuscular responses to SMT are mostly influenced by the rate of force application, while peak force modulation yields changes in the vertebral displacement. Rate of force application should therefore be defined in future studies. Clinical implications of various SMT dosages in patients with spine related pain should also be investigated.

**Trial registration:**

ClinicalTrials.gov NCT02550132. Registered 8 September 2015

## Background

**Fig. 1:**
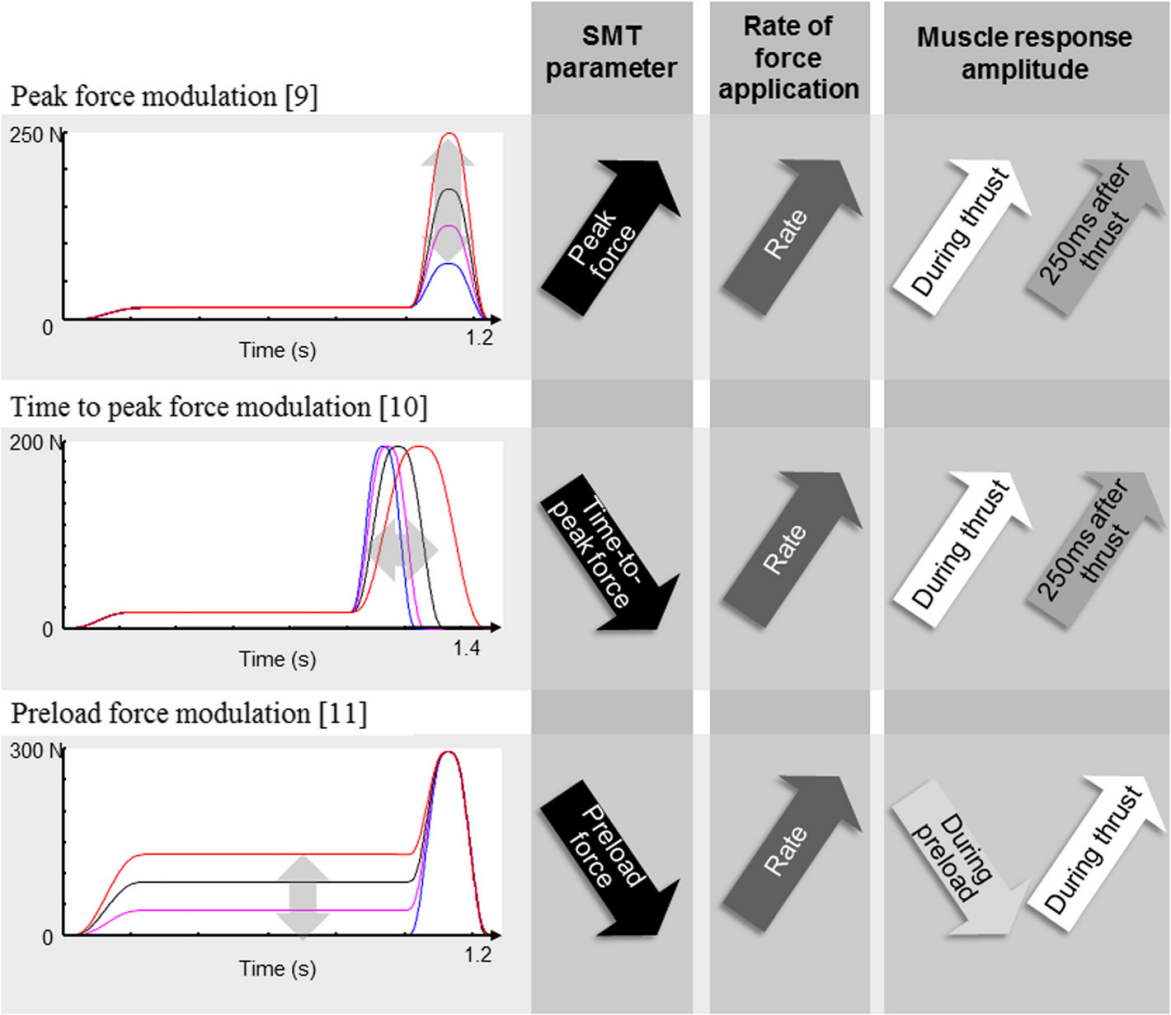
Modulation of SMT preload force, peak force, and time to peak force. These studies revealed an increase in muscle response amplitudes with increasing rate of force application by either increasing peak force, decreasing preload force, or decreasing time to peak force. SMT: spinal manipulation therapy

Spinal manipulation therapy (SMT) has been reported as a cost-effective therapy for spine related pain and is part of the therapeutic arsenal of numerous practitioners such as physiotherapists, chiropractors and osteopaths [1]. Although publications on SMT effectiveness have increased in the past years, evidences supporting the physiological mechanisms underlying its effects are scarce [2, 3]. Expertise in SMT has been associated with the capacity of controlling SMT biomechanical parameters (i.e. preload force, time to peak force, and peak force) [3, 4] and studies, mainly based on animal models or human cadavers, showed a dose-response relationship between these parameters and a few biomechanical and physiological response to SMT [5–7]. Considering the potential differences between animals, cadavers and humans responses [8], our research group has undertaken a series of experiments aimed at investigating neuromechanical responses to various SMT biomechanical parameters dosage in healthy humans through the use of an apparatus allowing the standardization of the SMT delivering. These studies showed that SMT yields different vertebral displacements and local muscles activity responses depending on the dosage of the SMT peak force [9], time to peak force [10], and preload force [11]. However, another parameter was also modulated in these experiments: the rate of force application (defined as the amount of force applied in a given period of time ((peak force-preload force)/time to peak force)). In our previous experiments, the rate of force application was indirectly modulated by modulating the peak force while keeping the time to peak force constant, modulating the time to peak force while keeping the peak force constant, and modulating the preload force while keeping the peak force and the time to peak force constant [9–11]. Results, illustrated in Fig. 1, showed that recorded muscular activities during and following the SMT thrust increased when the rate of force application increased. These observations are supported by recent animal studies reporting an increased muscle spindle discharge when rate of force applications are increased [6, 12].

Although there is indirect evidence that the rate of force application modulates neuromuscular responses through peak force or thrust duration modulations, such an assumption remains to be confirmed. Therefore, the objective of the present study was to determine if different SMT force-time profiles where a constant rate of force application would be maintained (through the modulation of the peak force and the time to peak force) lead to similar neuromechanical responses. Based on the available data relative to the effect of SMT biomechanical parameters modulation, it was hypothesized that neuromuscular responses would be similar across SMT force-time profiles, while vertebral displacements would increase as SMT peak force increases. A synthesis of the previous studies, clinical perspectives and the implication of these results in futures studies are also be discussed.

## Methods

### Participant sample

Twenty-five healthy volunteers with a mean age of 24.76 years (SD: 3.8) were recruited through advertisement on the University campus. A general screening of each potential participant was performed by an experienced chiropractor in order to assess for inclusion and exclusion criteria. Individuals presenting with thoracic or lumbar pain, history of back trauma or surgery, severe osteoarthritis, inflammatory arthritis, vascular conditions or any contraindication to the use of SMT were excluded. The study was submitted to, and approved by the Université du Québec à Trois-Rivières Human Research Ethics Committee (CER-12-181-06.37) and all participants provided their written informed consent.

### Experimental protocol

**Fig. 2:**
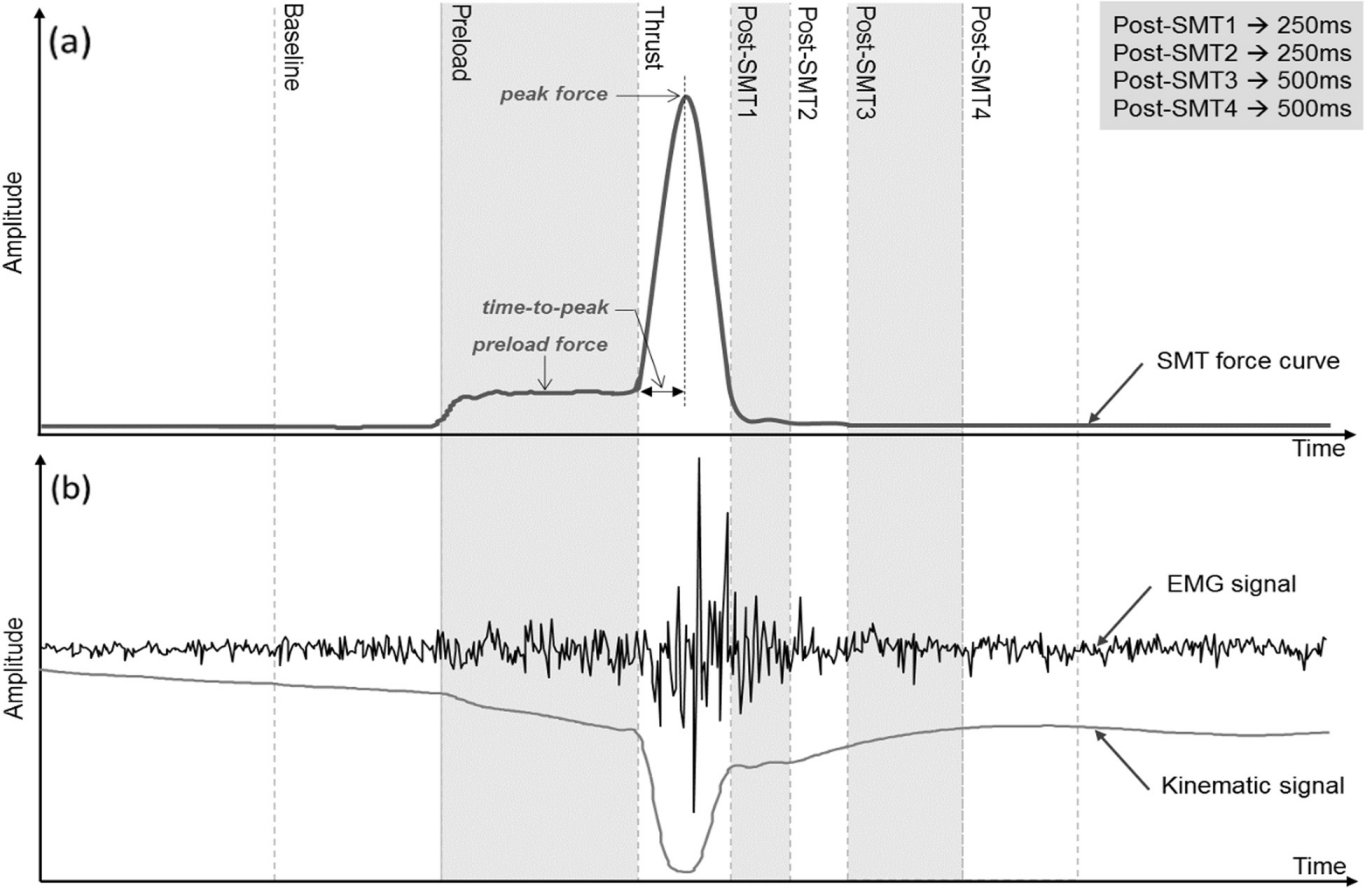
**a** Typical SMT force-time profile with time-windows, and (**b**) typical sEMG and kinematic responses. The rate of force application (N/s) is equal to the force applied (peak force – preload force) divided by the time to peak force. SMT: spinal manipulation therapy

The 45-min experimental session was conducted at the Université du Québec à Trois-Rivières Neuromechanics and Motor Control Laboratory. Before asking them to lie prone on a chiropractic table, a demonstration of the simulated SMT was first shown to each participant. Surface electromyographic (sEMG) electrodes and kinematic markers were then placed in order to record thoracic erector spinae muscles’ activity and vertebral displacements during each SMT. Each participant received four different SMT force-time profiles delivered at T7 vertebral level (see a typical SMT force-time profile in Fig. 2a). Those four SMTs presented the same preload force of 25 N and a similar rate of force application of 2200 (±8) N/s corresponding to previously published data on rate of force application used during SMT [13–15]. The SMTs differed in their time to peak force (ms) and peak force (N), respectively fixed as follow for each applied SMT force-time profile: (1) 57 ms/150 N, (2) 80 ms/200 N, (3) 102 ms/250 N and (4) 125 ms/300 N. To avoid any sequence effect, the four SMTs were randomized across participants through a predetermined association list of participant number and SMT sequence. A five-minute rest was taken between each trial.

### Neuromuscular and kinematic acquisition

Four sEMG electrodes (Model DE2.1, Delsys Inc., Boston, MA, USA) with a common mode rejection ratio of 92 dB at 60 Hz and an input impedance of 1015 Ω were used to record the paraspinal muscles activity. Following fiber muscle orientation, electrodes were applied over the thoracic spine erector spinae muscles on each side of the spine at approximately 2 cm of T6 and T8 spinal processes [16]. For each participant, the left acromion was chosen for the reference electrode. For each electrode location, the skin was gently shaved, then gently abraded with fine-grade sandpaper and finally wiped with alcohol swabs. Data were sampled at 1,000 Hz with a 12-bit A/D converter (PCI 6024E, National Instruments, Austin, TX, USA), collected by LabView (National Instruments, Austin, TX, USA) and processed by Matlab (MathWorks, Natick, MA, USA). A motion analysis system (Optotrak Certus; Northern Digital, Waterloo, Ontario, Canada) was used to perform the kinematic data acquisition at 100 Hz. Kinematic markers were placed on T6, T7 and T8 spinous processes.

### Apparatus

The four desired SMT force-time profiles were precisely simulated by an apparatus using a servo-controlled linear actuator motor (Linear Motor Series P01-48x360, LinMot Inc., Zurich, Switzerland) which vertically displaced a slider applied directly to the spine. The contact point between the apparatus and the spine was performed by a twin-tip padded rod on both T7 transverse processes. Based on a microcontroller, the target SMT force-time profile loaded from a computer was accurately reproduced by the linear motor. A close loop constantly compared the target force with the measured force and adjusted the intensity of the motor in order to obtain a measured force as close as possible to the targeted force. A complete technical description and details of safety features are provided by Descarreaux et al. [17].

### Data analysis

sEMG data were filtered digitally by a 20 to 450 Hz band-pass 4th order Butterworth filter. The power line interference was removed by applying notch filters at 60 Hz and its harmonics. Furthermore, electrocardiogram contamination of sEMG signal due to the electrodes position in the thoracic spine area was accurately cancelled by the method described in Nougarou et al. [18]. In order to analyse the muscular response according to SMT main events, seven time-windows were defined based on the SMT force-time profile as shown in Fig. 2a: “Baseline” of 500 ms duration to establish muscular activity before SMT, “Preload phase” of 750 ms, “Thrust phase” with a duration equal to twice the time to peak force, and four phases successively following the “Thrust phase” with two windows of 250 ms and two windows of 500 ms (referred as “Post-SMT1“, “Post-SMT2“, “Post-SMT3“ and “Post-SMT4“). For each trial, the four sEMG recordings were divided in seven normalized root mean square (RMS) values corresponding to each time-windows; the normalisation was achieved by dividing the obtained RMS by the RMS value of the “Preload phase”.

Regarding kinematic data, the absolute vertebral displacement from preload to thrust (maximal vertebral displacement in the “Thrust phase”) was computed for the three markers (T6, T7, and T8 level). The relative displacement of T6 with respect to T7 and of T8 with respect to T7 was also computed by subtracting the maximal displacement of T7 to the maximal displacement of T6 and T8, respectively. Figure 2b illustrates a typical EMG and kinematic response to SMT.

### Statistical analyses

All dependent variables (normalized RMS and absolute and relative displacement) were found to be normally distributed, and were submitted to analysis. The effect of the four SMT force-time profiles on the muscular activity were evaluated through repeated measures analysis of variance (ANOVAs) performed independently for each electrode during each define time-windows (“Preload phase”, “Thrust phase”, and post-SMT phases). Repeated measures ANOVAs were also computed to assess the SMT force-time profile effect on the absolute and relative vertebral displacements during the “Preload phase” and the “Thrust phase”. Whenever ANOVA yielded a significant effect, a Tukey post-hoc test was computed. Polynomial contrast analysis was used to test for the *a priori* hypothesis that the absolute vertebral displacement would linearly increase with SMT peak force. The level of statistical significance was set at *p <* .05 for all analyses and the STATISTICA statistical package version 10 (Statsoft, OK, USA) was used to perform the analyses.

## Results

### Effect of SMT force-time profiles on muscular activity

**Fig. 3:**
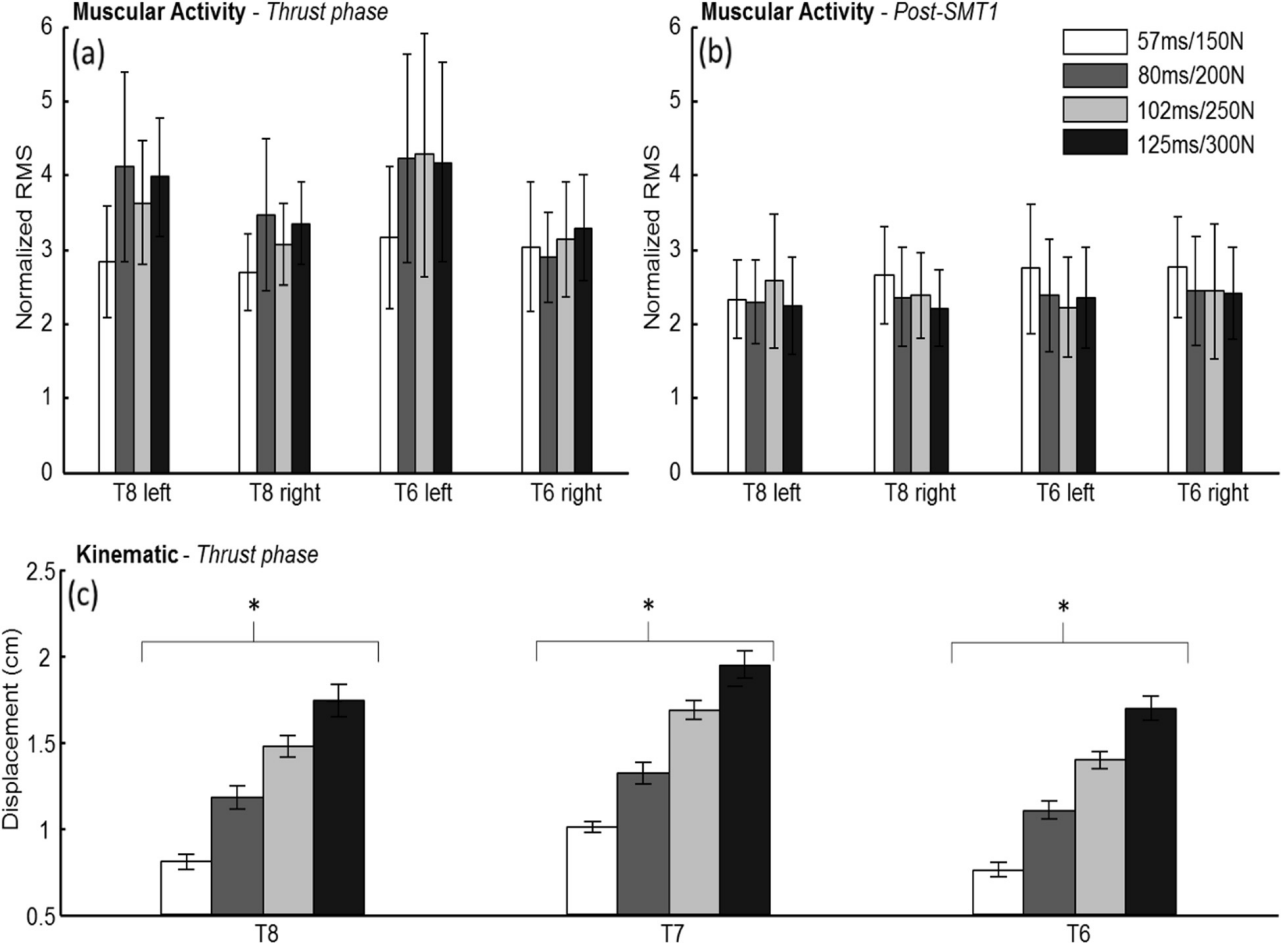
Normalized RMS during (**a**) “Thrust phase” and (**b**) “Post-SMT1”, and (**c**) absolute vertebral displacements during “Thrust phase”. Muscular activity values (normalized RMS) are presented for the four applied SMT force-time profiles and all electrodes. Vertebral displacements are shown for all markers and SMT force-time profiles.* refers to *p <* .05

A main effect of SMT force-time profiles on the muscular activity computed from sEMG signals was observed for the left T8 (*p =* .03) and T6 (*p =* .04) during the "Thrust phase". For these trials, muscular responses were significantly lower during the 150 N peak force condition (T8: mean ± SE = 2.83 ± 0.30 and T6: mean ± SE = 3.72 ± 0.68) than the 300 N peak force condition for T8 (mean ± SE = 4.81 ± 0.88) or the 250 N peak force condition for T6 (mean ± SE = 4.93 ± 0.91). No differences were observed for these electrodes during the other define time-windows or for the right T6 and T8 electrodes during all define time-windows. Neuromuscular response amplitudes for the “Thrust phase” and the “Post-SMT1” are shown in Fig. 3a, b. Details of normalized RMS values obtained during the “Thrust phase” and post-thrust time windows (“Post-SMT 1 to 4”) are presented in Table 1.

**Table 1:** Mean (SE) of normalized RMS values during “Thrust phase” and post-thrust time intervals (“Post-SMT 1 to 4”)

EMG	Time to peak force/peak force	Thrust phase	Post-SMT 1	Post-SMT 2	Post-SMT 3	Post-SMT 4
T8 Left	57 ms/150 N	2.83 (0.30)	2.60 (0.34)	1.22 (0.15)	0.89 (0.10)	0.73 (0.08)
	80 ms/200 N	4.12 (0.50)	2.54 (0.33)	1.13 (0.17)	0.85 (0.12)	0.70 (0.11)
	102 ms/250 N	4.03 (0.52)	2.58 (0.36)	1.18 (0.15)	0.86 (0.11)	0.74 (0.11)
	125 ms/300 N	4.80 (0.88)	2.24 (0.26)	1.14 (0.18)	1.02 (0.20)	1.03 (0.24)
	F(3,72)	F = 3.23, *p =* .03^a^	F = 0.79, *p =* .50	F = 0.17, *p =* .92	F = 0.76, *p =* .52	F = 2.13, *p =* .10
T8 Right	57 ms/150 N	3.51 (0.84)	2.64 (0.26)	1.38 (0.15)	1.05 (0.11)	0.88 (0.09)
	80 ms/200 N	3.46 (0.41)	2.36 (0.27)	1.20 (0.16)	0.91 (0.09)	0.75 (0.08)
	102 ms/250 N	3.57 (0.55)	2.38 (0.23)	1.15 (0.15)	0.92 (0.09)	0.82 (0.09)
	125 ms/300 N	3.79 (0.49)	2.20 (0.21)	1.25 (0.18)	0.96 (0.13)	0.88 (0.14)
	F(3,72)	F = 0.13, *p =* .94	F = 1.41, *p =* .25	F = 0.64, *p =* .52	F = 0.66, *p =* .58	F = 0.67, *p =* .57
T6 Left	57 ms/150 N	3.72 (0.68)	3.03 (0.45)	1.14 (0.11)	1.02 (0.21)	0.74 (0.08)
	80 ms/200 N	4.75 (0.76)	2.69 (0.43)	1.12 (0.13)	0.83 (0.09)	0.64 (0.08)
	102 ms/250 N	4.92 (0.91)	2.56 (0.44)	0.91 (0.08)	0.75 (0.07)	0.64 (0.07)
	125 ms/300 N	4.17 (0.53)	2.35 (0.27)	0.94 (0.14)	0.85 (0.16)	0.87 (0.21)
	F(3,72)	F = 2.94, *p =* .04^a^	F = 0.45, *p =* .72	F = 2.26, *p =* .09	F = 0.72, *p =* .54	F = 1.21, *p =* .31
T6 Right	57 ms/150 N	3.03 (0.35)	2.76 (0.27)	1.23 (0.12)	0.91 (0.09)	0.74 (0.08)
	80 ms/200 N	3.13 (0.33)	2.43 (0.29)	1.10 (0.11)	0.89 (0.10)	0.77 (0.09)
	102 ms/250 N	3.40 (0.40)	2.43 (0.36)	1.26 (0.24)	0.92 (0.14)	0.85 (0.15)
	125 ms/300 N	3.87 (0.64)	2.67 (0.36)	1.22 (0.23)	0.93 (0.12)	0.87 (0.12)
	F(3,72)	F = 0.92, *p =* .43	F = 0.45, *p =* .72	F = 0.22, *p =* .88	F = 0.04, *p =* .99	F = 1.21, *p =* .61

^a^Statistically significant

### Effect of SMT force-time profiles on vertebral displacements

As shown in Fig. 3c and Table 2, modulation of the SMT force-time profile led to significant differences in absolute vertebral displacements during the “Thrust phase” for all markers (all *p* values < .001) but not during the “Preload phase”. The polynomial contrast analysis for linear trend was significant for the contacted vertebrae (*F* (1, 17) = 354.80, *p <* .001) and both adjacent vertebras (T6: *F* (1, 12) = 104.71, *p <* .001 and T8: *F* (1, 19) = 468.68, *p <* .001). Vertebral displacements increased in average (SD) of 0.92 cm (0.02) between the 57 ms/150 N and the 125 ms/300 N SMT force-time profiles. In addition, analysis of vertebral displacements relative to the contacted vertebrae (T7) led to significant differences when modulating the SMT force-time profile for the T6 marker during the “Thrust phase” only (*F* (3, 42) = 2.91, *p =* .045).

**Table 2:** Mean (SE) of vertebral displacements (cm) during “Preload phase” and “Thrust phase”

Markers	Time to peak force/Peak force	Preload Phase	Thrust Phase
T8	57 ms/150 N	0.64 (0.07)	0.76 (0.04)
	80 ms/200 N	0.59 (0.05)	1.10 (0.05)
	102 ms/250 N	0.60 (0.06)	1.40 (0.05)
	125 ms/300 N	0.60 (0.05)	1.70 (0.07)
	F	F(3, 72) = 0.73, *p =* .66	F (3, 36) = 73.33, *p <* .001^a^
T7	57 ms/150 N	0.67 (0.06)	1.01 (0.03)
	80 ms/200 N	0.64 (0.05)	1.32 (0.06)
	102 ms/250 N	0.66 (0.05)	1.69 (0.05)
	125 ms/300 N	0.65 (0.06)	1.40 (0.08)
	F	F(3, 72) = 0.16, *p =* .92	F (3, 51) = 201.14, *p <* .001^a^
T6	57 ms/150 N	0.49 (0.06)	0.81 (0.04)
	80 ms/200 N	0.45 (0.05)	1.18 (0.07)
	102 ms/250 N	0.50 (0.04)	1.48 (0.06)
	125 ms/300 N	0.51 (0.05)	1.74 (0.09)
	F	F(3, 72) = 0.53, *p =* .54	F (3, 57) = 241.32, *p <* .001^a^

^a^Statistically significant

## Discussion

The results of the present study showed an increase in vertebral displacements when increasing SMT peak forces were applied, which is in accordance with previous studies using animal models [19–21]. However, there was no progressive increase in neuromuscular responses related to increasing SMT peak force when the rate of force was kept constant. These results suggest that vertebral displacements during SMT are mostly modulated by SMT peak force, since a previous study showed that modulation of SMT time to peak force does not significantly affect vertebral displacements in healthy humans [10]. This is also supported by a Colloca et al. [21] study that showed that posterior to anterior vertebral displacement responses linearly increased with increasing mechanical force (maintaining a constant pulse duration = 100 ms).

Few studies evaluating the rate of force application effect can be found in the literature, but the present one can be compared to studies where the preload force, the time to peak force or the peak force were modulated. Reed et al., and Pickar et al., using animal models, found that increasing the rate of force application (by increasing the force applied [12, 22], decreasing the thrust duration [12, 23] or decreasing the preload force [6]) led to an increase in mean instantaneous discharge frequencies of muscle spindles, with a maximal response at rates greater than 300 N/s. As mentioned in the introduction, the present study design did not, however, compare changes in rate of force application, since it had previously been established that increasing the force applied to the spine [9], decreasing the preload force [11] or decreasing the time to peak force [10] yielded increased neuromuscular responses. Instead, it was decided to keep the rate of force application constant by modulating the peak force and the time to peak force to test the assumption that a constant SMT rate of force application would yield similar neuromechanical responses.

The initial hypothesis was partly supported since the sagittal vertebral displacement during the posterior to anterior SMT increased with peak force (even if the rate of force application was kept constant by increasing the time to peak force and keeping the preload force constant). In a recent study [11] where the preload force was modulated, changes in the sagittal vertebral displacement were minimal, despite the changes in the rate of force application (decreasing the preload force led to an increase in the rate of force application), and seemed to be conditioned by the total force applied during SMT rather than the rate of force application.

Overall, these results, combined with previous studies’ results, suggest that biomechanical responses to SMT are mostly modulated by the total amount of force applied to the spine (force applied during preload + force applied during the thrust), whereas neuromuscular responses seem to be mostly affected by the rate of force application. Rate of force application, once a certain threshold is reached, seems to trigger similar neuromuscular responses. This holds true for peak forces of 200 to 300 N and changes in neuromuscular responses may be observed at higher force levels.

In order to better study the relationship between the preload force and the rate of force application, a study where the preload force is manipulated while the rate of force application is kept constant should be conducted.

## Strength and limitation

Although it has been previously reported that modulation of SMT parameters yields different neuromechanical responses, the present study is the first to assess these responses in humans while keeping a constant rate of force application. However, only healthy individuals were evaluated, thereby results may not reflect how patients with spinal related pain react to SMT, and the relationship between these responses and changes in clinical outcomes is not known.

### Clinical implication

Since the present study showed that increasing SMT peak force while keeping a constant rate of force application generates increased vertebral displacements without modifying muscle response amplitudes, the modulation of these parameters based upon the expected goal should be considered by clinicians. Indeed, avoiding an increase in spinal stiffness, by the modulation of the rate of force application, might be important in patients with muscle spasms. However, further studies are needed to confirm the clinical implication of the present results.

## Conclusion

The present study suggests that the neuromuscular response to SMT is influenced by the rate of force application, while modulation of SMT peak force yields changes in biomechanical parameters such as the vertebral displacement. Since rate of force application can be modulated through the other SMT biomechanical parameters, this parameter should be defined in future studies. Clinical implications of various SMT dosages in patients with spine related pain also need to be investigated.

## Abbreviations

ANOVA, analysis of variance; RMS, root mean square; sEMG, surface electromyography; SMT, spinal manipulation therapy
